# Database limitations for studying the human gut microbiome

**DOI:** 10.7717/peerj-cs.289

**Published:** 2020-08-17

**Authors:** Camila K Dias, Robert Starke, Victor S. Pylro, Daniel K. Morais

**Affiliations:** 1 Departament of Biochemistry, Universidade Federal do Rio Grande do Sul, Porto Alegre, Rio Grande do Sul, Brazil; 2 Institute of Microbiology of the Czech Academy of Sciences, Prague, Czech Republic; 3 Department of Biology, Universidade Federal de Lavras - UFLA, Lavras, Minas Gerais, Brazil

**Keywords:** Human Microbiome, Database, Gut microbiome, Functional diversity, KEGG, Uniprot, EggNOG

## Abstract

**Background:**

In the last twenty years, new methodologies have made possible the gathering of large amounts of data concerning the genetic information and metabolic functions associated to the human gut microbiome. In spite of that, processing all this data available might not be the simplest of tasks, which could result in an excess of information awaiting proper annotation. This assessment intended on evaluating how well respected databases could describe a mock human gut microbiome.

**Methods:**

In this work, we critically evaluate the output of the cross–reference between the Uniprot Knowledge Base (Uniprot KB) and the Kyoto Encyclopedia of Genes and Genomes Orthologs (KEGG Orthologs) or the evolutionary genealogy of genes: Non-supervised Orthologous groups (EggNOG) databases regarding a list of species that were previously found in the human gut microbiome.

**Results:**

From a list which contemplates 131 species and 52 genera, 53 species and 40 genera had corresponding entries for KEGG Database and 82 species and 47 genera had corresponding entries for EggNOG Database. Moreover, we present the KEGG Orthologs (KOs) and EggNOG Orthologs (NOGs) entries associated to the search as their distribution over species and genera and lists of functions that appeared in many species or genera, the “core” functions of the human gut microbiome. We also present the relative abundance of KOs and NOGs throughout phyla and genera. Lastly, we expose a variance found between searches with different arguments on the database entries. Inferring functionality based on cross-referencing UniProt and KEGG or EggNOG can be lackluster due to the low number of annotated species in Uniprot and due to the lower number of functions affiliated to the majority of these species. Additionally, the EggNOG database showed greater performance for a cross-search with Uniprot about a mock human gut microbiome. Notwithstanding, efforts targeting cultivation, single-cell sequencing or the reconstruction of high-quality metagenome-assembled genomes (MAG) and their annotation are needed to allow the use of these databases for inferring functionality in human gut microbiome studies.

## Introduction

High-throughput sequencing (HTS) of DNA allows for the comparative analyses of diversity, abundance, important functional genes and their traits, without the need of cultivating individual microbes and at far greater depths than ever before (Weinstock, 2012). Full functional annotation is limited to those organisms that have undergone isolation and extensive characterization while the vast majority were not yet studied and the annotation is based on the similarity to the genomes of very few studied model organisms (Pham & Kim, 2012). However it is important to point out that the view over this paradigm has been changing (Martiny, 2019). HTS as cultivation–independent techniques brought benefits to the microbiome field by expanding our knowledge on many environments as, for instance, the human gut microbiome (Goodman et al., 2009; Bella et al., 2013). The steps of DNA extraction, library preparation and sequencing of DNA became a routine in laboratories and companies in the last twenty years after the sequencing of the first bacterial genome (Fleischmann et al., 1995; Loman & Pallen, 2015). Currently, it is possible to generate an extraordinary amount of data and to correlate this information with healthy and unhealthy conditions of the human body (Cryan & Dinan, 2012; Shreiner, Kao & Young, 2015). However, the available experimental and bioinformatics methods leave space for bias and unreliable results (McLaren, Willis & Callahan, 2019). Well-established HTS methods can present high sequencing error rates, which could lead to wrongful assignment of taxonomy. Besides the issues with the methodology, there are also issues with database updates considering the ever-growing amount of data (Song, Lee & Nam, 2018). Another issue that has to be dealt with when researching a given microbiota is the choice of binning approach, a group of methods that can be used to cluster contigs into what would be representative of a single population genome. This step is considered a bottleneck in metagenomics studies since everything that is inferred about a certain study depends on the sensitivity of the binning methodology. The results obtained with binning methodologies are not always trustworthy, and this can lead to taxonomic and functional associations that do not resemble reality (Strous et al., 2012).

The quality of the inferred functions in a microbial community depends on the presence of similar sequences in the database used for comparison. According to the assessment of taxonomical and functional profiles in microbial ecology studies, the analysis suffers from theoretically unresolvable arbitrariness and ambiguities mainly because there is not a correct scale to be used when comparing the taxonomy and the functions associated to a microbiome (Inkpen et al., 2017).

In 2010, Qin and colleagues described the attainment of 576.7 gigabases of DNA sequences in a study with 124 subjects, which resulted in 3.3 million non-redundant microbial genes, as a part of the MetaHIT (Metagenomics of the Human Intestinal Tract) project (Erlich & The MetaHIT Consortium, 2010). Most of these sequences were found to be from bacterial origin, some of them from archaea, with very little representation of eukaryotes and viruses. From all microbial species found in the study, the authors estimate that 160 are shared between most of the subjects (Qin et al., 2010).

The relationship between taxonomy and function is becoming of utmost importance in microbiome studies since the establishment of the Koch’s postulates (Koch, 1982; Neville, Forster & Lawley, 2018). Therefore, large metagenome projects such as the Human Microbiome Project have deepened our understanding about the functional composition in the human gut to the level of describing ten core pathways in this environment (Lloyd-Price et al., 2017). Inferences that establish a relationship between microbiome profiles and their associated functions are often achieved through the cross referencing of different databases, combining and validating the shared information (Park et al., 2010; Huerta-Cepas et al., 2016; Franzosa et al., 2018).

In this work, we used the entries from Uniprot Knowledge Base (Uniprot KB) that were cross-referenced to the Kyoto Encyclopedia of Genes and Genomes Database (KEGG Database) and to the Evolutionary genealogy of genes: Non-supervised Orthologous Groups (EggNOG Database). The UniprotKB is recognized as one of the largest protein repositories available (The Uniprot Consortium, 2017). It contains more than 100 cross-referenced databases and combines entries curated manually (Swissprot) and entries that were automatically annotated without review (TrEMBL). Currently only 12% of the database entries have gone through curated annotation (The Uniprot Consortium, 2017). The KEGG database is among the world’s most used biological databases and helps to connect sets of genes to high-level functions through the grouping of genes in KEGG Orthologies (KO) (Kanehisa et al., 2015). The KOs supply the link between pathways and modules. KOs are organized in four levels of functional information from the first more broad to the fourth and more specific level. This system allows that experimental evidence of a function obtained from a specific organism to be extended to another one that has a gene associated to that same KO (Kanehisa et al., 2015; Kanehisa et al., 2018; Mao et al., 2005). A different database for functional characterization for inferred orthologous groups is EggNOG, which uses two parallel approaches of summarizing known attributes of group members and determines which annotations can be robustly propagated to the group as a whole. This database is also prominent in the field, focusing on the formation and identification of orthologous groups (OGs) instead of function, as in KEGG Database. The EggNOG pipeline provides the prediction of orthologous groups, being applied a hierarchical consistency algorithm, in addition to a function annotation pipeline (which includes information from the KEGG Database), phylogenetic analysis and pairwise orthology prediction (Huerta-Cepas et al., 2016). Even though both databases revolve around orthology predictions based on sequences, the different approaches make the information provided by them complementary.

Thus, for this study, we applied a cross reference search in Uniprot KB and the deepest level of KEGG Orthology or EggNOG databases.

Considering the aspects raised here about high-throughput DNA sequencing in human microbiome studies, we seek to answer how well some databases would be able to depict an observed human gut microbiota. We hypothesize that, although the scientific community considers these databases gold standards for studying proteins and their functions throughout the tree of life, they might not be suitable for evenly representing the set of bacteria associated to the human gut microbiota. 

It is known that amplicon-based studies have high descriptive power regarding the composition of microbial communities. Hence, two of the three studies used here for the production of a list of species common to the human gut employed this methodology (Eckburg et al., 2005; Yatsunenko et al., 2012). Here we highlight some other studies in the field that have very similar human gut microbiota sets to the one we used as reference for our study. Table S1 demonstrates the similarities and differences found between our study and the ones here cited (David et al., 2014; Zhernakova et al., 2016; Chauhan et al., 2018; Johnson, 2020). We then added a shotgun sequencing-based study linked to the MetaHIT project, which was one of the first big attempts to describe the human gut microbiome (Qin et al., 2010) to increase the data types included in the list.

Finally, these data were used to evaluate the presence of commonly detected gut prokaryotes in the Universal Protein Knowledge Base (UniprotKB) under the functional classification of KEGG Orthologs and EggNOG.

## Materials & Methods

### Data mining

Microbial communities from three publications were chosen to represent the gut microbiota on the level of bacterial species (Eckburg et al., 2005; Qin et al., 2010; Yatsunenko et al., 2012). For the generation of the species list, strains and other subclassifications were not considered.

The first study chosen is among the pioneers human gut microbiome studies, where the methods included amplification of the 16S rRNA gene, using broad bacterial and archaeal primers, cloning and bidirectional sequencing (Eckburg et al., 2005).  Three healthy adult subjects’ samples were used in this study including mucosal tissue and fecal samples. The second study is associated to the MetaHIT project (Erlich & The MetaHIT Consortium,, 2010) and included samples from 124 subjects healthy, obese or with inflammatory bowel disease, from Europe. The method used was the shotgun metagenome sequencing through Illumina GA II (Qin et al., 2010). Finally, the last article comprised a target sequencing of the 16S rRNA gene (V4 region) of 531 subjects’ samples from Venezuela, Malawi and the United States (Yatsunenko et al., 2012), using the Illumina HiSeq 2000 DNA sequencing platform.

### Database entries download and cross-referencing

The Uniprot Database (The Uniprot Consortium, 2017) entries were downloaded using the browser on the 27th of September 2019, containing the parameters “Entry”, “Entry name”, “Status”, “Protein names”, “Gene names”, “Organism”, “Length”, “Cross reference (KO)”, “Cross reference (EggNOG)”, “Phylum”, “Genus”, “Species”, “Kingdom” and “Superkingdom”. We limited the entries downloaded to those with functional affiliation in either KEGG or EggNOG, aiming to evaluate how well Uniprot Database would be capable of describing the functions associated to a certain list of species from the human gut microbiome. Firstly, the Uniprot entries were filtered for Bacteria and Archaea, and these entries were then compared to the species list retrieved from the three publications.

The lists and the identifiers for KOs and NOGs resulting from the cross search of the species and genus list produced from the articles and the Uniprot entries were filtered and sorted until data was suitable for plotting.

### Visualization

The RStudio interface version 1.1.453 was used for the making of barplots and histograms (R Studio Team, 2016).

### Data access

The scripts used for assessing and comparing the cross-reference from UniprotKB and KEGG or EggNOG together with the species and genus list obtained from the publications (Eckburg et al., 2005; Qin et al., 2010; Yatsunenko et al., 2012) is available as Files S1 and S2. The EggNOG database was updated to version 5.0.0 (Huerta-Cepas et al., 2019) in 2019, and in this work we used data from the version 4.5.1 as this is the current version used by Uniprot. The raw data can be downloaded from FigShare DOI: 10.6084/m9.figshare.12555422 and 10.6084/m9.figshare.12555425.

## Results

The complete list of species contained 131 different species and 52 different genera (Table S1). The KEGG database provided information on 40 genera and 53 species whereas the EggNOG Database provided information on 47 genera and 82 species. It is important to highlight that the list of species produced here did not intend to mimic with perfection a human gut microbiome sample, but simply present species and genera reported to have been found in the three representative studies, and that are also well represented in other four evenly time distributed studies (Table S1). When comparing our list of genera with newer studies (David et al., 2014; Zhernakova et al., 2016; Chauhan et al., 2018; Johnson, 2020), representing 6 years of technological evolution, we found that only 19 new genera were included (Table S2), this result tells us that the addition of more studies wouldn’t change our outcomes and also, that there is need to direct database update effort on unknown taxa as, the newer studies brought many genus that were already in our list and were lacking in the current databases.

After filtering out the Uniprot entries not belonging to Bacteria or Archaea, and removing those without functional annotation, we got 6,531,071 entries for KOs and 4,749,622 entries for EggNOG IDs. The Uniprot database contains two columns for searching species or genus name, the “TAXONOMY” column and the “ORGANISM” column. From the entries cross-referenced with KOs, 819,541 matched the genera list when comparing to the “TAXONOMY” column and 776,714 entries when comparing to the “ORGANISM” column. About the species list there were 245,067 entries cross-referenced between Uniprot and KEGG when comparing the “TAXONOMY” column and 244,451 entries when comparing to the “ORGANISM” column. As for the EggNOG Database, 821,946 entries cross-referenced with Uniprot entries when comparing the genera list to the “TAXONOMY” column and 794,084 entries when comparing to the “ORGANISM” column. The entries that cross-referenced between EggNOG and Uniprot using the species list were 297,298 when comparing to the “TAXONOMY” column and 297,940 when comparing to the “ORGANISM” column. However, part of these entries belonged to genera or species that had its taxonomical identification changed over time. Thus, we made a table to present the differences in annotation encountered in both cross-reference searches. For KEGG results 40 genera presented the same names as in our articles list, 12 were not represented by KEGG Database and 27 genera had its taxonomy updated in the database and, sometimes, one genus was split into two, in this case, both genera were included, increasing the number of recovered genera to 67. For species, 53 were present in our list and on KEGG with the same taxonomical identification, while 78 species from our list were not represented by the database and 3 species presented different identifications, increasing the number of recovered species to 55. The EggNOG Database presented entries on 47 genera with the same names as in our list, being that 5 genera were not represented by EggNOG and 18 genera presented different taxonomical identification. The cross-reference search of Uniprot-EggNOG against our species list did not hit species with taxonomical updates, being that 82 out of 131 were found (Table S3). These taxonomical identifications discrepancies are due to the fact that when species and genera names undergo some taxonomic update, the databases use the most recent name followed by the previous name in brackets, hence these different names came along in our cross-search output. Another source of confusion over the genera names, is that some genera had its taxonomy hierarchy changed, becoming more than one genus.

We proceeded the analysis focusing on the results provided by the cross-reference using the “TAXONOMY” column from EggNOG and KEGG. We made this choice based on having more KOs or EggNOG codes for the “TAXONOMY” searches. Considering that each entry may have multiple KOs or EggNOG codes and that the same species may have different entries with the same code, in total, the unique entries for the species search in KEGG returned 5,668 KO numbers, and the entries for the genera search returned 6,788 KO numbers. For the EggNOG search, we obtained 54,523 EggNOG codes for the species search, being that 1,383 were arCOGs (Archeal Clusters of Orthologous Genes), 3,889 were COGs (universal with best coverage for Bacteria Clusters of Orthologous Groups) and 49,251 were ENOGs (protein single entry).

**Figure 1:**
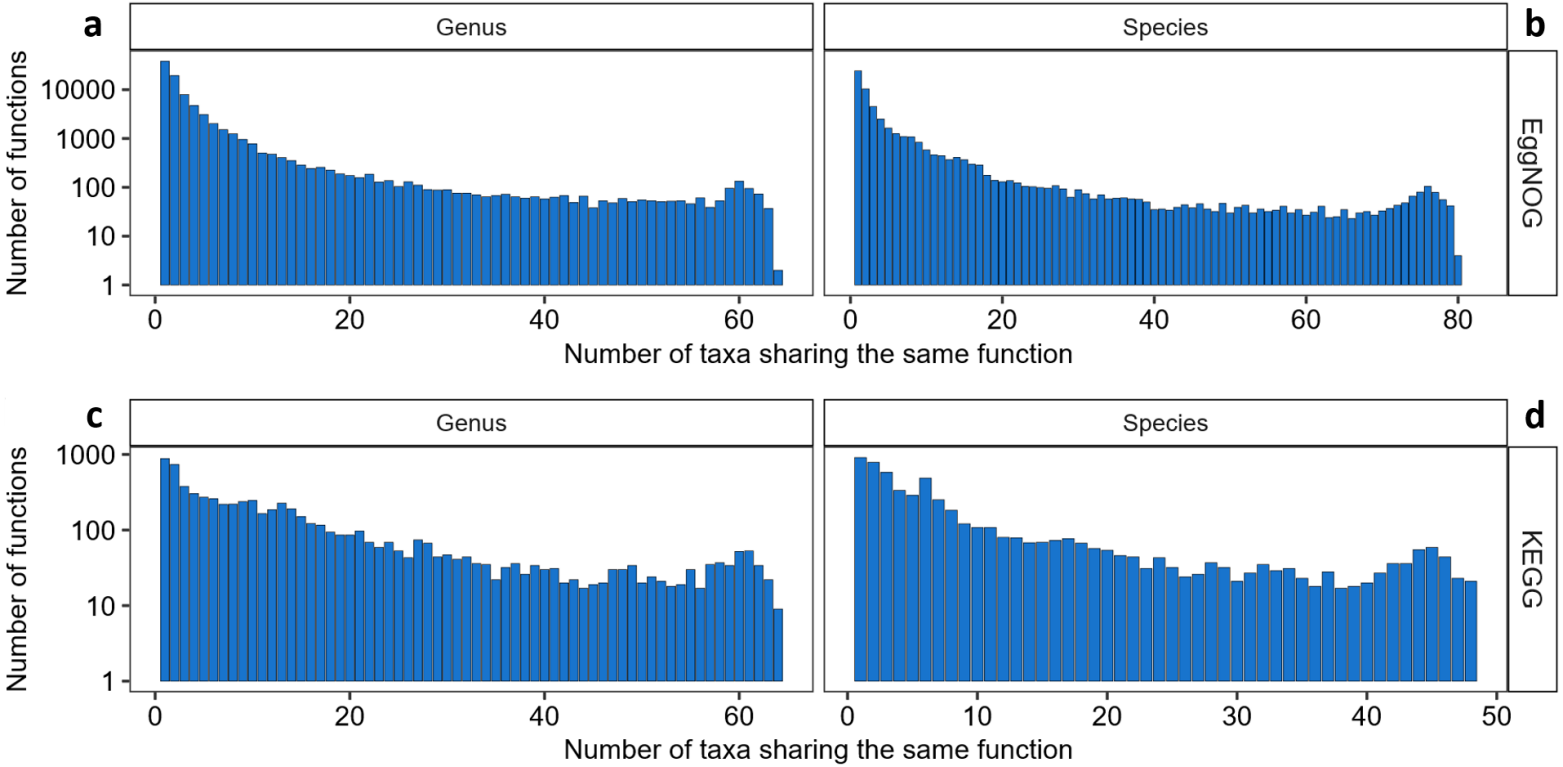
The relationship between the number of taxa sharing NOGs and KOs. (A) Number of genera sharing EggNOGs, (B) number of species sharing EggNOGs, (C) number of genera sharing KOs and (D) number of species sharing KOs.

We then proceeded to analyzing the KOs and EggNOG function distribution throughout the genera and species contained in the list. As depicted in Fig. 1 it is clear that EggNOG presented an overall higher amount of functions, but only presenting one genus and one species with more than 1,000 functions associated. Even though KEGG presented a smaller number of functions associated to one or more genera and species, KEGG was more homogeneous than EggNOG, in its distribution of taxa sharing the same functions.

It is interesting to observe that almost 24% of all KOs only appear in one or are shared by a maximum of two genera and around 76% (5,182 KOs) of all KOs are shared by twenty or less genera, approximately one third of the genera sharing the same KOs (64). Furthermore, 31 KOs were shared by 64 and 63 genera, (Fig. 1C), meaning that no KO was shared by all the genera evaluated and these KOs were the ones that were shared by most of the genera. A table with the functions associated to these 31 “core KOs” is available as Table S3. Amongst these KOs, are functions related to purines and pyrimidines metabolism, the pentose phosphate pathway, aminoacyl-tRNA biosynthesis pathway, DNA mismatch repair and mainly ribosomal proteins. Within these 31 most common KOs we also observed one KOs associated with peptidoglycan synthesis and, consequently, resistance to Vancomycin.

Furthermore, the distribution of KOs shared by species was similar to the distribution of KOs shared by genera, as depicted in Fig. 1D. Almost 30% of all KOs are unique to some species or are shared by two species (1698 KOs) and around 87% of all KOs are shared by 24 or less species, which stands for half of the maximum of species sharing the same KOs (48). From the 44 “core KOs” shared by 48 and 47 species (Table S3), 21 KOs are correspondent to ribosomal proteins and fourteen are enzymes related to glycolysis, gluconeogenesis, fructose, galactose and mannose metabolism, aminoacyl-tRNA biosynthesis, amino acids metabolism and purines and pyrimidines metabolism. Some other functions such as cell cycle proteins, quorum sensing and transporter proteins are represented by one or two KOs. Incidentally, the same KO that had relation to Vancomycin resistance present in the genera core KOs is present in the species core KOs. The details of function and protein names for each KO entry can be viewed in this link as Supplementary Table S3.

Regarding the distribution of EggNOG codes throughout genera and species there was higher amount of functions shared by species and genera when compared to KEGG as depicted in Fig. 1. EggNOG Database provided more functional codes for the same protein than KEGG, and the distribution of functions shared by taxa was small. Over 44% of all EggNOG codes were unique, being found only once among all genera and almost 98% of all codes are shared by 32 or less genera, (Fig. 1A), including the unique ones. The core EggNOGs for the genera cross-search are represented by 39 codes shared by 64 and 63 genera (Table S3). From the core NOGs found in our cross-search for 64 and 63 genera some functions worth highlighting are: translation, post-translation modification, transcription, coenzyme transport and metabolism, nucleotide transport and metabolism, intracellular trafficking, cell cycle control, energy conversion and DNA replication. Again, the most recurrent function was ribosomal proteins, totalizing 15 out of the 39 core NOGs for the species cross-search. The species cross-reference with EggNOG showed that almost 45% of NOGs are unique to a single species and almost 97% of all NOGs are shared by 41 or less species (Fig. 1B). Amongst the functions present in our core NOG list for the species cross-search we highlight transcription, post-translational modification, replication, nucleotide transport and metabolism, cell wall and membrane biogenesis and carbohydrate transport and metabolism. As in the genus search, the majority of codes present in the core species NOG list belong to proteins associated to translation and ribosomal proteins (26 out of 48) (Table S3). Interestingly, only one NOG was present in all 82 species represented by the EggNOG cross-search with UniprotKB, it is a chaperonin associated to the function of preventing misfolding and promoting refolding.

**Figure 2:**
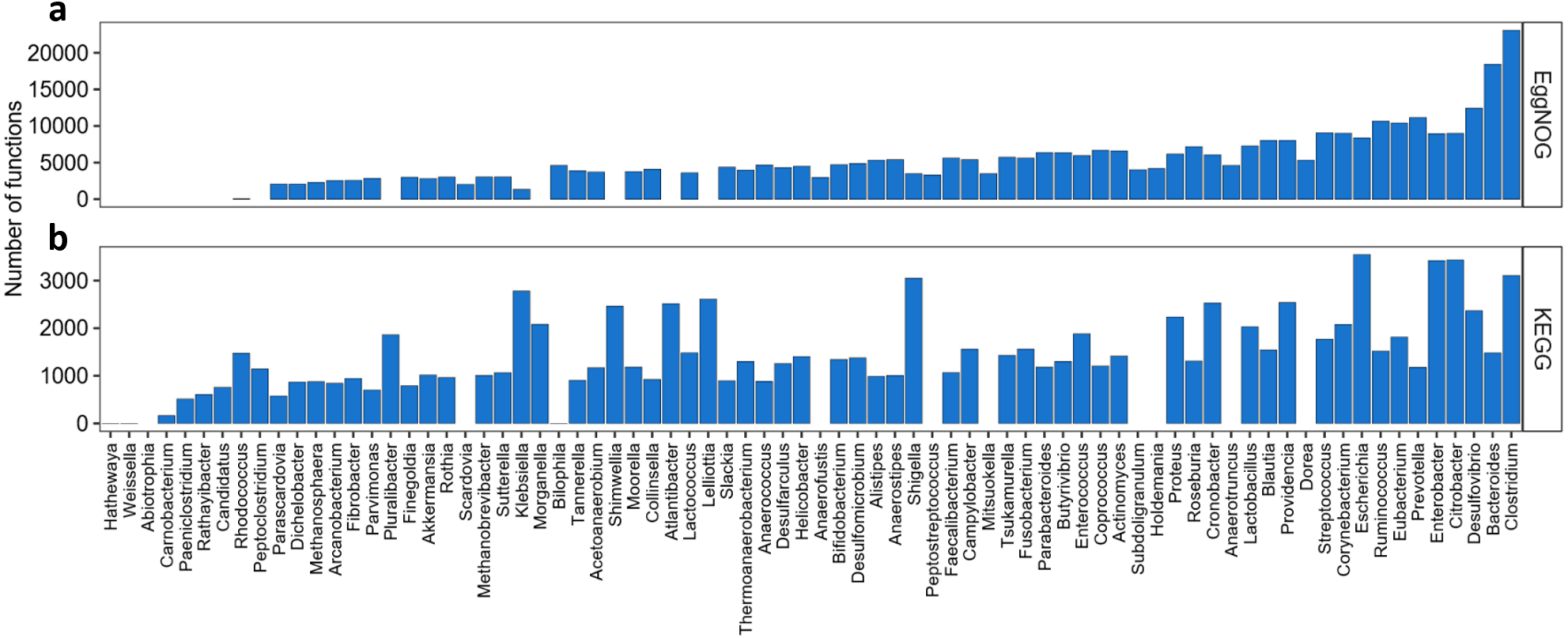
Abundance of KOs and NOGs throughout different genera detected in UniprotKB. (A) EggNOG couts and (B) KEGG counts. Genera are ordered by the sum of identifiers in both databases. Some genera only had identifiers in one database.

Once knowing which functions where best described by the KO and NOG numbers, another question to answer is which genera and species would be best represented by this set of KOs or NOGs, and which ones would not. As shown in Fig. 2, we found 67 genera in the KEGG-Uniprot cross-search which matched, our list, considering the taxonomical updates. The EggNOG-Uniprot cross-search resulted in 65 genera. Some genera are much better represented than others (Fig. 2). *Clostridium* was the genus best described by the Uniprot database with 23,081 entries. After *Clostridium,* the genera*Bacteroides, Desulfovibrio, Prevotella, Ruminococcus and Eubacterium* also had more than 10,000 KOs associated to them. As for EggNOG, the genus *Clostridium* was among the ones presenting the highest number of NOGs associated (3,108), although the most covered genus was *Escherichia*, having 3,546 unique entries. Other genera which presented high numbers of NOGs associated were *Citrobacter*, with 3,435, *Enterobacter*, with 3,420, and *Shigella*, with 3,052.

**Figure 3:**
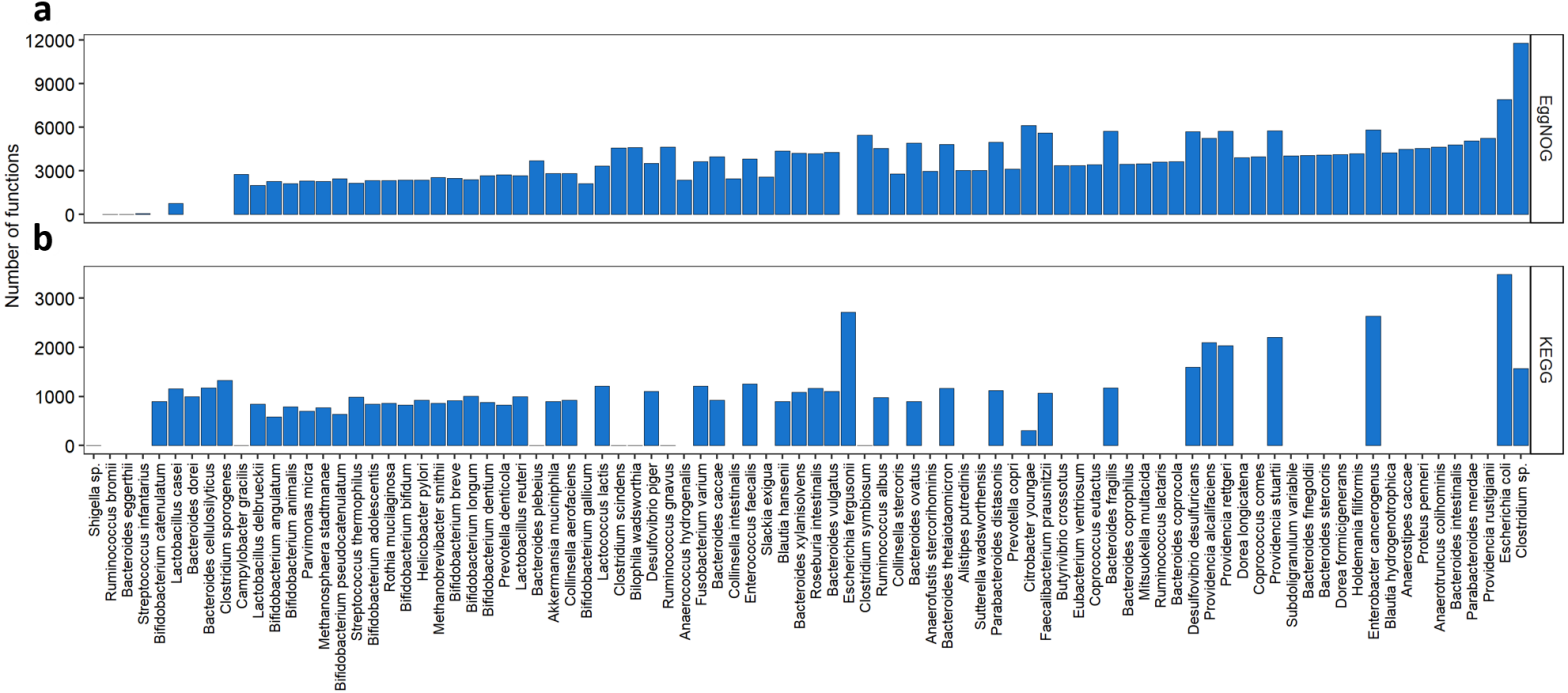
Abundance of KOs and NOGs throughout different species detected in UniprotKB. (A) EggNOG counts and (B) KEGG counts. Species are ordered by the sum of identifiers in both databases. Some species only had identifiers in one database.

We also wanted to evaluate how the KOs and NOGs were distributed over the species, as shown in Fig. 3. Taking into account all species resulting from our search, 55 species had Uniprot entries and KO numbers available, 82 species presented Uniprot entries and EggNOG numbers. From those 55 species for the KEGG search, 7 had very low number of KOs associated to them (one to six KOs per species), differently from all the others that had at least 300 associated KOs. Interestingly, many of those species were strains of *Shigella sp.* The species with the highest number of KOs associated with, were *Escherichia coli* (3,488 KOs)*, Escherichia fergusonii, Enterobacter cancerogenus, Providencia stuartii, Providencia alcalifaciens* and *Providencia rettgeri* having more than 2000 KOs each. From the 82 species for the EggNOG search, only three species presented less than 700 NOGs associated. These were *Streptococcus infantarius* with 58 NOGs, *Bacteroides eggerthii* with 12 NOGs and *Ruminococcus bromii* with 2 NOGs. One the other hand, 13 species presented more than 5,000 NOGs. The best described species were *Escherichia coli* (7,908 NOGs), followed by *Citrobacter youngae* (6,113 NOGs) and *Enterobacter cancerogenus* (5,830 NOGs).

As seen in Fig. 3 some species such as *Bacteroides plebeius* had only one KO associated to it. In this specific case the corresponding KO (K20830) is a beta-porphyranase [EC: 3.2.1.178], described as a function identified only in the Japanese gut microbiota and related to the acquisition of genetic information by *Bacteroides plebeius* from marine bacteria (Hehemann et al., 2010). The only KO associated to *Campylobacter gracilis* (K02111) is related to the ATPase subunit alpha [EC:7.1.2.2 and EC: 7.2.2.1]. Seven different strains of *Shigella sp.* presented only one KO attributed (K18767) a beta-lactamase gene class CTX-M [EC:3.5.2.6]. This new class of beta-lactamases seems to have been acquired by *Shigella* species by horizontal transferring from *Enterobacteriaceae* species (Bradford, 2001). As to the EggNOG cross-search one species *Ruminococcus bromii* presented only two NOGs associated to it, both regarding a chaperonin protein. Another species presented a small number of NOGs associated (12), *Bacteroides eggerthii*, and the functions related to those NOGs were post translational modification, protein turnover and chaperones; replication, recombination and repair; leucine biosynthesis; and transcription.

**Figure 4:**
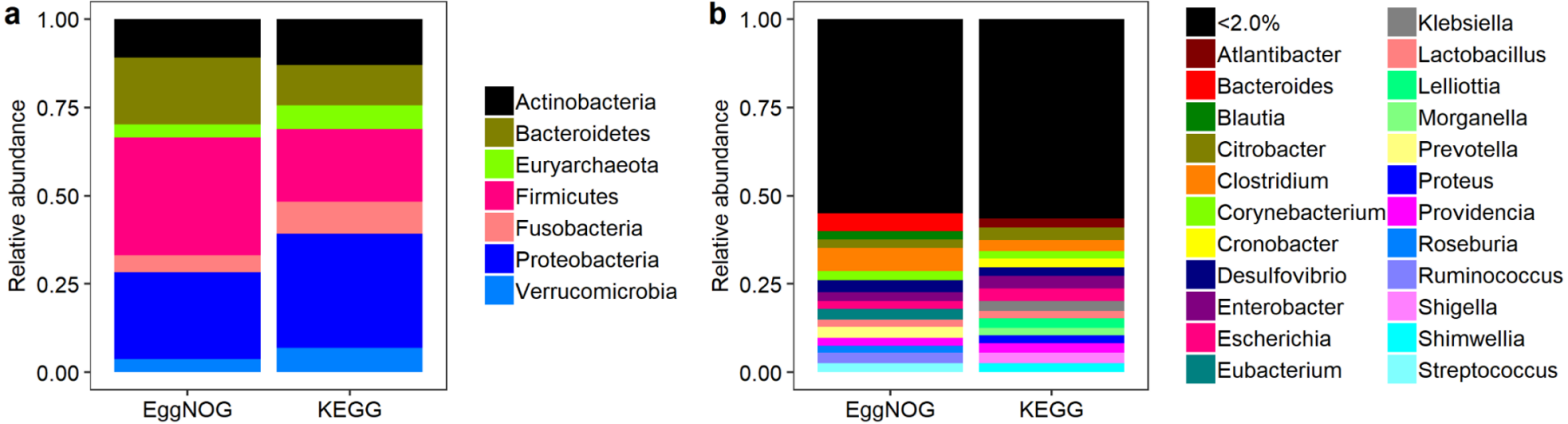
Relative abundance of NOGs and KOs throughout phyla (A) and genera (B) for EggNOG and KEGG cross-searches.

Moving over to phyla distribution, as seen in Fig. 4A the *Proteobacteria* phylum presented the highest coverage of KOs, with a total of 4,276 . In the same sense *Firmicutes* presented higest abundance of NOGs, with a total of 25,538 As shown in Fig. 4B the relative abundance of codes throughout genera was considerably different between KEGG and EggNOG. It appears that the KEGG Database provided a more homogenous result for the genera cross-search and the number of genera was very similar between KEGG and EggNOG searches. In fact, the EggNOG Database presented an overall higher number of codes associated to genera. All the raw hit tables, containing the counts of KOs and NOGs per species, genera and phyla, as well as, the number of species presenting a specific entry, are compressed in a file as File S1.

Furthermore, knowing that some species or genera were better represented than others, we wondered if some of the best-represented species belong to the best-represented genera in each database search. If this was the case we would hypothesize that the information collected in the genera search is not so dissimilar from the information collected in the species search. However, that was not the case for the EggNOG cross-search. As an example, we point out that, the second best described species was *Escherichia coli* and, in the genus list, *Escherichia* was the 11th best described. Another example is *Citrobacter youngae*, which was the 3rd best described species belonging to the genus *Citrobacter* which was the 9th best described genus. This representation discrepancies show that when a cross-search is conducted using only genera information the yield of functions and annotations associated to them can be very different from what would have come up on a species search.

Lastly, we highlight the percentages of KOs and NOGs associated to the Bacteria or Archaea domains. For the KEGG cross-search, taking into account the species list, almost 7% (897) of all KOs belonged to the Archaea domains, therefore more than 93% belonged to the Bacteria domain. As for the EggNOG cross-search almost 4% (2,789) of all NOGs belonged to the Archaea domain and almost 96% belonged to the Bacteria domain. Due to the higher number of NOGs resulting from the EggNOG cross-search it appears that its representation of the Archaea domain is lesser than the KEGG Database when, in fact, there were almost three times more NOGs associated to the Archaea domain than KOs.

## Discussion

This study intended to evaluate how well a cross reference study utilizing the Uniprot KB and the KEGG or the EggNOG databases could describe a mock human gut microbiome community. The databases were chosen considering their importance on the microbiome field and respectability associated. After joining the species lists from each of the mentioned articles (Eckburg et al., 2005; Qin et al., 2010; Yatsunenko et al., 2012), we were able to confirm that the articles shared very few or none of the same species, which could be viewed as a diverse assessment of the human gut microbiome. The genera list was then based on the genera present in this species list.

The lack of entries from 78 species in the KEGG database and 49 species in the EggNOG database, using the Uniprot Database shows that even though new sequencing techniques have made possible the attainment of a great amounts of data, data processing and database increase is not up to the same speed. The mismatch between data generation and database update become more evident if we take into account that the species lists used here were generated from articles published up to more than 7 years ago. The EggNOG database had, generally, better coverage of the taxa in our mock human gut microbiome. The EggNOG database presented almost 10 times more entries associated to our species list than KOs from the KEGG database. EggNOG entries were more numerous in reason of their

We have chosen to search the database using the taxonomic affiliation instead of using sequence similarity, to avoid the biases and limitations of homology inference. The taxonomic information could be found in two fields of Uniprot, one called “Organism” and the other “Taxonomy”. These two didn’t always gave the same names, sometimes the entries had the name of the genus within brackets, meaning it was necessary to add other steps to the analysis, which was to check for repeated genus names with or without brackets, other times, the entries presented family names in the species category, and genera names that did not match the first name of the species, which also made the analysis more laborious, as such “bugs” had to be sorted individually.

Regarding the KO distribution per genus and per species, some examples of genera and species were highlighted, presenting unexpected numbers of KOs. An extreme case is the *Bilophila* genera with only the KO K03851 associated to it, which is correspondent to taurine-pyruvate aminotransferase (EC: 2.6.1.77), and the article associated to the entry is from the year 2000 (Laue & Cook, 2000). Consequently, the same happened to the species *Bilophila wadsworthia*, which has only one KO associated to it, as seen in Fig. 3. Other interesting extreme cases are the species that presented a single KO or two linked to it, as mentioned previously in the results. This shows a lag between the information being generated and the annotation about a certain species being available, also shows the unbalanced accumulation of data over the same species, or even genus. The EggNOG cross-search did not present as much genera and species with so little information related to them. Only one genus, *Abiotrophia*, presented 2 NOGs and the species *Ruminococcus bromii*, *Bacteroides eggerthii* and *Streptococcus infantarius* presented 2, 12 and 58 NOGs, respectively. Conversely, this difference between EggNOG and KEGG can be due to EggNOG’s organization and the fact that this database works with different hierarchical organization levels. EggNOG is organized in

A disadvantage brought by the lack of evenness in database entries might be that, sometimes, the data obtained can be deceiving. For example, in Fig. 2 the genus *Escherichia* presents the highest number of KOs and the genus *Clostridium* has the highest number of NOGs, in this way, one could imagine that many species from this genus contributed to this high number of KOs. That is not exactly the case for KEGG, since all the KOs found for this genus were coming from the high amount of functions described for *E. coli* and this phenomena might not only drive hits to this species, but also lose important information from other species of the same genus, while other genera get much less information, for instance *Bilophila*, *Weissella* or *Hathewaya*. However, it was the case for EggNOG, where *Clostridium sp.* (6 strains) was the species with the highest number of NOGs associated as the genus Clostridium. Other *Clostridium* species, such as *Clostridium symbiosum* and *Clostridium scindens*, also presented considerable NOG numbers, moreover, Escherichia also had 2 species representatives in the EggNOG database. In this sense it appears that the EggNOG database would have a broader coverage for studying the human gut microbiome.

## Conclusions

Regarding the comparison of KEGG and EggNOG databases performances while being cross-referenced with the Uniprot database, overall, the EggNOG database presented better results to the parameters evaluated in this work concerning a representation of the human gut microbiome, created from the species and genera identified in the studies of Eckburg et al. (2005), Qin et al. (2010) and Yatsunenko et al. (2012). The EggNOG database showed better representation of the list of species and genera from the mock human gut microbiome. It also presented more EggNOG entries associated to each genus and species. However, it has to be taken into account that the organization of EggNOG can be one of the reasons for the much higher total of entries between EggNOG and KEGG.

Nonetheless we observed a general lack of representativeness and evenness of KOs and EggNOG entries associated to species and genera of the human gut microbiota. Although there are powerful methods to infer homology and assign functionality through sequence comparison, such methods are limited by the sequences that have been deposited in the databases. The lack of more than 50% of the species used in this study for KEGG cross-reference and 37% for EggNOG cross-reference indicates that part of the human gut microbial diversity is left to be described by inference-based methods applied to distant relatives. Moreover, low representation of multiple species or genera expose the imbalance of the database. This information helps us to find target taxa that will bring unique information from cultivation, single-cell genome studies or genomes recovered from metagenomes.

##  Supplemental Information

10.7717/peerj-cs.289/supp-1 Supplemental Information 1 List of species used to inspect the UniprotKB

10.7717/peerj-cs.289/supp-2 Supplemental Information 2 Evaluation of the sampling effort We selected four new studies to see how many new genera would be included if we kept sampling studies through out the years.

10.7717/peerj-cs.289/supp-3 Supplemental Information 3 Core KOs and ENOGs The most shared identifiers from the KEGG and EggNOG database, we used those identifiers which were present in the maximum number of species and genera.

10.7717/peerj-cs.289/supp-4 Supplemental Information 4 Script for processing KEGG data

10.7717/peerj-cs.289/supp-5 Supplemental Information 5 Script for processing EggNOG data

## Additional Information and Declarations

### Data Availability

The following information was supplied regarding data availability: The raw data and code are available as Supplemental Files and at Figshare: Kumazawa Morais, Daniel (2020): uniprot-database_(type_eggnog).27.09.2019.tab.rar. figshare. Dataset. https://doi.org/10.6084/m9.figshare.12555425.v1. and Kumazawa Morais, Daniel (2020): uniprot-database_(type_ko).27.09.2019.tab.rar. figshare. Dataset. https://doi.org/10.6084/m9.figshare.12555422.v1.
